# Nonhuman Adenoviral Vector-Based Platforms and Their Utility in Designing Next Generation of Vaccines for Infectious Diseases

**DOI:** 10.3390/v13081493

**Published:** 2021-07-29

**Authors:** Marwa Alhashimi, Ahmed Elkashif, Ekramy E. Sayedahmed, Suresh K. Mittal

**Affiliations:** Immunology and Infectious Disease, and Purdue University Center for Cancer Research, Department of Comparative Pathobiology, Purdue Institute for Inflammation, College of Veterinary Medicine, Purdue University, West Lafayette, IN 47907-2027, USA; alhashim@purdue.edu (M.A.); aelkashi@purdue.edu (A.E.); esayedah@purdue.edu (E.E.S.)

**Keywords:** adenoviral vector, nonhuman adenoviral vector, viral vector, vaccine, infectious disease

## Abstract

Several human adenoviral (Ad) vectors have been developed for vaccine delivery owing to their numerous advantages, including the feasibility of different vector designs, the robustness of elicited immune responses, safety, and scalability. To expand the repertoire of Ad vectors for receptor usage and circumvention of Ad vector immunity, the use of less prevalent human Ad types or nonhuman Ads were explored for vector design. Notably, many nonhuman Ad vectors have shown great promise in preclinical and clinical studies as vectors for vaccine delivery. This review describes the key features of several nonhuman Ad vector platforms and their implications in developing effective vaccines against infectious diseases.

## 1. Introduction

Adenoviruses (Ads) have been an invaluable tool for in vivo gene delivery, which has dramatically influenced the usefulness of Ad vectors for vaccine development and gene therapy. Ad vector platforms have numerous advantages for vaccine applications, including immunogen expression for a short duration, low incidence of Ad genome integration into the host DNA, development of balanced humoral and cell-mediated immune (CMI) responses, and a good established safety profile. Currently, the most studied Ad is human Ad type 5 (HAd5); however, less prevalent HAds including types 26 and 35 as well as several nonhuman Ads, including chimpanzee Ad, canine Ad, bovine Ad, ovine Ad, porcine Ad, avian Ad, and others, are being explored as vectors for gene delivery.

### 1.1. Advantages and Utility of Ad-Based Vectors

The Ad-based platforms have been utilized as gene delivery systems for many applications, owing to the widely studied Ad biology, which unraveled many advantageous properties for the virus [1]. The Ad is a non-enveloped virus with a linear double-stranded DNA genome that can easily package a large transgene cassette. The virus uses a wide-range tissue tropism, allowing for gene expression in different target cells and tissues. It is reasonably straightforward to grow Ad to very high titers using basic cell culture practices [2]. Moreover, Ad possesses a high transduction efficiency, allowing the vector to express its cargo gene in dividing and non-dividing cells. The Ad is safe for the target host due to the low incidence of insertional mutagenesis. In general, several wild type Ads are associated with asymptomatic to limited pathogenesis in humans; however, their safety profile is further increased by making them replication-defective.

Ad-based vectors are used to deliver and express the desired transgenes in host cells. The carried transgene can be a therapeutic gene for gene therapy or an antigen transgene for vaccines. For gene therapy applications, Ad vectors have at least two major drawbacks—a transient expression nature of the transgene, and the immunogenicity of the vector’s backbone, thereby reducing the duration of transgene expression. However, these drawbacks of Ad vectors are advantageous for vaccine applications.

### 1.2. Ad-Induced Innate Immunity

Due to the high immunogenicity of Ads, strong innate and adaptive immune responses are induced in an infected host. The process is initiated with the recognition of pathogen-associated molecular patterns (PAMPs). Pathogen recognition receptors (PRRs) found on the surface of the host cells identify PAMPs, and an innate immune response cascade is activated. It is initiated with the secretion of pro-inflammatory cytokines, which trigger the differentiation of immature dendritic cells. An Ad may activate the immune system by triggering multiple defense mechanisms, including activation of blood factors and elimination of virus-containing cells with the help of cytotoxic immune cells [3]. During viral entry in the blood vessel, the Ad vector interacts with several blood factors [4,5]. The coagulation factor X (FX) complexed with Ad could initiate Toll-like receptor (TLR) 4 (TLR4) signaling. Tripartite motif-containing factor 21 (TRIM21) recognizes intracellular antibodies and activates interferon regulating factor (IRF) signaling pathways [5]. TRIM21 functions by recruiting antibody-bound Ad and targets it to the proteasome. This leads to rapid disassembly of the virus in the cytosol before translation of its genomic material can occur. The complement component’s intracellular sensing activates inflammatory cascades and tumor necrosis factor (TNF) receptor-associated factors [6]. Intravenous (i.v.) inoculation of mice either with HAd5 or HAd2 resulted in rapid complement activation [7].

In a context where the virus is left unrecognized by blood factors, it can be recognized during the attachment to the cell surface receptor. Ad fiber protein on the capsid plays a primary role in binding to specific cell receptors for virus entry [8]. Additionally, β3 integrin is important for IL-1α expression during Ad cell entry [9]. IL-1α signaling activates chemokine expression through the IL-1R1, attracting polymorphonuclear cells to the sites of infection [10,11]. However, in the liver, Kupffer cells can eliminate the HAd by trapping them for suicidal necrotic cell death [12]. Finally, HAd DNA can be identified in the cytoplasm by the cyclic GMP-AMP synthase (cGAS) pathway [13] which is then converted into cyclic guanine adenine monophosphate (cGAMP) [14]. Due to the absence of this pathway, IFN stimulation is significantly lower in the liver cells. Cellular DNA damage response mechanisms can also identify HAd double-stranded DNA (dsDNA) in the nucleus. The host defense system can break this viral dsDNA [15]. However, the HAd genome encodes two proteins, E4-ORF3 and E1B-55K, preventing the viral DNA damage response [16].

### 1.3. Basic Features of Ad Vector-Based Vaccines

The development of viral vector-based vaccines requires a delicate balance between identifying a delivery platform that stimulates the production of a robust and durable immune response without posing a significant risk to those being vaccinated. This challenge is enhanced in the case of high-consequence viral pathogens. In addition, the use of live-attenuated vaccines is fraught with the risk of potential reversion to wild type or pathogenic for immunocompromised individuals. Subsequently, considerable effort has been put toward developing vaccines that are safe and provide adequate immunity. Currently, recombinant Ad vectored vaccines are widely used in many preclinical and clinical studies. By stimulating both TLR-dependent and TLR-independent pathways, Ad-based vaccines could mount balanced humoral and CMI responses against many infectious diseases [17,18].

Replication-competent Ad (RC-Ad) vectors are designed by deleting the early (E) region 3 (E3) and have been used as vaccine delivery systems without significant risks [19]. However, replication-defective (RD-Ad) vectored vaccines with deletions of the E1 and E3 regions are generally effective and safe [20]. In addition, a new single-cycle Ad (SC-Ad) vector was designed, which retains the genes required for DNA amplification and vaccine antigen gene expression, but without the genes needed for functional progeny. This type of vaccine can express the antigen similar to RC-Ads [21] and up to 300-fold more than RD-Ad [21,22,23]. However, SC-Ads do not benefit from a second wave of infection like RC-Ads, so they may not be as potent as RC-Ad vectored vaccines in humans.

### 1.4. Preexisting Ad Vector Immunity

**Figure 1:**
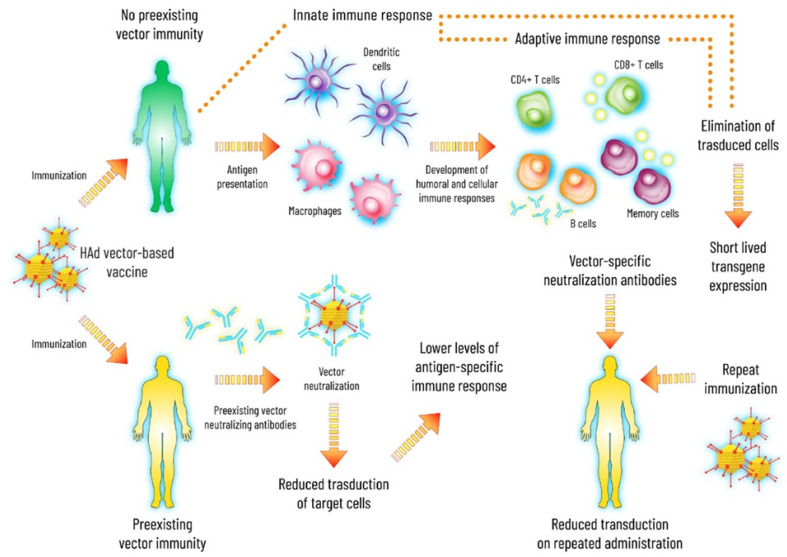
Consequences of preexisting adenovirus (Ad) vector immunity. Preexisting Ad vector immunity inhibits initial transduction with Ad vectors and, therefore, impedes the first use of many Ad vectors. In individuals with no preexisting Ad immunity, the first use of Ad vector would elicit strong innate, cellular, and humoral immune responses. The vector-specific cellular immunity then eliminates the majority of the transduced cells while the vector-specific humoral immunity blocks subsequent infection with the same Ad vector.

A significant challenge that impedes the success of several HAd vector-based vaccines in clinical trials is the high prevalence of preexisting immunity due to the presence of over 100 types of Ads in humans. Thereby, most individuals are exposed to one or more Ad types during their lifetime, leading to the development of Ad-specific immunity. Similar situations also occur in several animal hosts. Ad-neutralizing antibody response is known as preexisting Ad vector immunity in relation to Ad vector-based gene delivery. The components of immune systems such as type I IFN-activated NK cells, Ad-neutralizing antibodies (nAbs), and Ad-specific T cells contribute to reducing several HAd vectors’ efficacy due to cross-neutralizing activities. Therefore, high levels of preexisting Ad immunity will adversely impact Ad vector-based gene delivery effectiveness (Figure 1).

Various approaches have been developed to circumvent preexisting immunity, including the use of rare HAd types as delivery vectors. The process of selecting rare HAd types is carried out by paying less attention to cross-reactive Ad-specific T cells and concentrating more on the absence of cross-neutralizing Ad antibodies in a population. In addition, several nonhuman Ads based on chimpanzee Ad, canine Ad, bovine Ad, ovine Ad, porcine Ad, avian Ad, and others have been developed as vectors to supplement or replace HAd vectors to evade preexisting vector immunity (Table 1). Furthermore, avoiding Ad-specific tissue-resident T cells can be the basis for testing different routes of immunization [24]. Thus, the development of nonhuman Ad vectors is a vital step toward regulating preexisting immunity, thereby developing more efficient vectors for vaccines and gene therapy.

## 2. Chimpanzee Ad Vectors

Chimpanzee Ad (ChAd) vectors possess many advantages, making them a promising platform for developing novel vaccines [25]. ChAd can be quickly grown in a human cell line, HEK293, to very high titers [26]. Additionally, the seroprevalence of ChAd is significantly lower than that of HAd5 in humans [27]; therefore, cross-neutralization due to preexisting immunity can be easily prevented. Importantly, ChAd can induce both B and T cell immune responses similarly to HAd5 [28,29] even in the presence of preexisting vector immunity [30]. However, the efficiency of immunogenicity varies for different types of ChAds [31].

ChAds are classified into six groups (A–F) based on the viral hexon protein [26]. However, among them, C and E were the most prevalent (90%), whereas only 9% B type was found [32]. Most of the ChAd vectors are generated by replacing the E1 region of the genome with a transgene. In addition, to overcome safety issues and/or increase transgene insertion capacity, E3 and/or E4 genes are also deleted [33,34]. Other changes were also explored to improve in vitro productivity by manipulating small non-coding RNAs [35].

### 2.1. ChAd63

A low seroprevalent ChAd that belongs to group E was developed as a replication-deficient ChAd vectored vaccine [26,36]. The preclinical assessment of heterologous prime-boost with ChAd63 and modified vaccinia virus Ankara (MVA) bearing the ME-TRAP antigen, a fusion protein consisting of a multi-epitope (ME) string fused to *Plasmodium falciparum* pre-erythrocytic thrombospondin-related adhesion protein (TRAP), in nonhuman primates showed a median response of 869 IFN-γ-producing spot-forming cells (SFCs) per million peripheral blood mononuclear cells (PBMCs) in response to ME after priming with the ChAd63 vaccine. Following the booster with the MVA vector, the response reached 5256 IFN-γ-producing SFC/million PBMCs, leading to robust protection [37]. Phase I and IIa clinical studies of prime-boost with ChAd63-ME-TRAP and MVA-ME-TRAP showed a good safety profile. Doses up to 2 × 10^11^ virus particles (VP) were well tolerated in healthy adults. A lower dose of 5 × 10^10^ VP of ChAd63-ME-TRAP induced ME-TRAP-specific INF-γ secreting CD8^+^ and CD4^+^ T cells [38]. This response was boosted with MVA-ME-TRAP to high levels, reaching 2000 SFC/million PBMCs, and induced high anti-ME-TRAP IgG levels lasting up to 9 months post-boost. This vaccine could provide sterile to partial protection in controlling malaria, including heterologous strains [39]. In another study, two antigens, apical membrane antigen 1 (AMA1) and merozoite surface protein 1 (MSP1), from blood-stage malaria were expressed in ChAd63 or MVA and tested alone or in combination with ME-TRAP [40,41]. Priming with ChAd63 expressing either antigen was superior to other forms of the vaccine (DNA and fowlpox). When boosted with MVA, there was an induction of higher levels of CD8^+^ and CD4^+^ T cell responses and anti-AMA1 or anti-MSP1 IgG antibodies. A phase I study (NCT03084289) explores novel routes of administration (intradermal and intravenous) for enhanced CMI responses.

### 2.2. ChAd3

ChAd3 vectors have been evaluated in preclinical and clinical trials as vaccines for Ebola virus (EBOV) and hepatitis C virus (HCV) [29,42,43,44,45,46]. Many studies have previously linked the induction of broad and sustained CD4^+^ and CD8^+^ T cell responses to better control of HCV [47,48]. To test this hypothesis, Folgori and colleagues designed a unique target immunogen (NSmut) based on conserved sequences that span nonstructural genes (NS3-NS5B), from b and BK genotypes of HCV, a region that houses well-established CD4^+^ and CD8^+^ T cell epitopes [49]. To overcome the preexisting vector immunity, the replication-defective ChAd3, which is serologically distinct and has a lower seroprevalence, was evaluated in a prime-boost regimen with MVA expressing NSmut in healthy human volunteers in a phase I clinical trial (NCT01070407) [46]. Intramuscular (i.m.) priming with ChAd3-NSmut induced HCV-specific CMI responses, including CD8^+^ and CD4^+^ T cell responses, inducing 1000 SFCs/10^6^ PBMCs. Following the MVA vaccine boost, there were increases in both the breadth and durability of induced T cells, reaching 7000 SFCs/10^6^ PBMCs one week after the boost. T cell responses were sustained up to 70–73 weeks post-boost. Moreover, ChAd3-NSmut/MVA-NSmut prime-boost could induce a cross-reactive T cell response against heterologous viral genotypes but at a reduced magnitude [50].

The preclinical assessment of a monovalent replication-defective ChAd3 expressing the Zaire EBOV glycoprotein (GP) (cAd3EBO) or bivalent ChAd3 vaccine expressing GP from Zaire EBOV and Sudan EBOV with the MVA vector carrying the GP gene was conducted as a prime-boost strategy in rhesus macaques [43]. A single i.m. injection with monovalent or bivalent vaccine provided complete protection against lethal EBOV challenge at 3 weeks post-immunization. Immunized animals showed a dose-dependent increase in GP-specific antibodies and CD8^+^ T cells. Boosting with the MVA vector after 8 weeks provided uniform protection up to 10 months post-boost against EBOV lethal challenge, while partial protection was achieved with a single dose of ChAd3 vector [43]. In a dose-escalating phase I clinical trial, the safety and immunogenicity of cAd3-EBO were evaluated in 20 healthy participants. A single i.m. injection of the cAd3-EBO vaccine of up to 2 × 10^11^ VP did not induce any adverse effects. At 4 weeks post-vaccination, GP-specific antibodies were developed with a geometric mean titer of 2037, GP-specific CD8^+^ T cells in 7 out of 10, and CD4^+^ T cells in 10 out of 10 of the participants, and these responses stayed up to 48 weeks post-vaccination [29]. Another phase I clinical trial of a prime-boost with ChAd3/MVA bearing the EBOV Zaire GP gene resulted in increased anti-GP antibody titers by 12-fold and GP-specific CD8^+^ T-cells by 5-fold, which persisted for 6 months post-boost. Neutralizing antibodies were detected in all study participants boosted with MVA [45]. In a randomized, placebo-controlled phase II clinical trial of cAd3-EBO-Z a dose of 1 × 10^11^ VP by the i.m. route to healthy participants conferred significant anti-GP IgG antibodies, and GP-specific CD4^+^ and CD8^+^ T-cell responses, which persisted up to 1 year post-vaccination [44].

### 2.3. ChAdOx1

ChAdOx1 is based on the ChAd Y25 isolate and has gained a lot of interest in recent years [51]. The human trial with ChAdOx1 NP+M1 expressing the influenza nucleoprotein (NP) and matrix protein 1 (M1) showed its safety and tolerability at doses up to 2.5 × 10^10^ VP. A single dose ChAdOx1 NP+M1 induced a robust CMI response [52]. When the same vector was boosted with the MVA vector expressing the same antigens, higher levels of humoral and CMI responses were detected and persisted for 18 months post-boost [53].

The ChAdOx1 platform was also explored for an HCV vaccine expressing a conserved domain covering multiple HCV genotypes. The immunization of mice elicited both broad and cross-reactive CMI responses as indicated by 1174 SFU/10^6^ PBMCs [54]. In addition, a ChAdOx1 vector expressing mycobacterial antigen 85A (Ag85A) was found to be well tolerated and immunogenic in humans [55], and the immunogenicity was further improved as a part of the BCG-ChAdOx1 85A-MVA85A prime-boost regime [56]. A single i.m. dose of ChAdOx1 MERS expressing the full-length spike (S) protein of the Middle East respiratory coronavirus (MERS-CoV) protected against MERS-CoV disease in a nonhuman primate model [57]. The ChAdOx1 vaccine (ChAdOx1-nCoV-19) containing the S gene of severe acute respiratory syndrome coronavirus 2 (SARS-CoV-2) was developed similar to the ChAdOx1-based vaccine for MERS-CoV [58]. Initial immunization and challenge studies in mice and rhesus macaques demonstrated the development of both humoral and CMI responses, conferring protection from coronavirus disease 2019 (COVID-19) following challenge with SARS-CoV-2. The ChAdOx1-nCoV-19 vaccine was evaluated for its safety and immunogenicity in phase I, phase II, and phase III trials (NCT04324606) [59]. A phase III trial with a two-dose i.m. immunization elicited S-specific humoral and CMI responses. There were high titers of neutralizing antibodies in 91% of participants after a single dose and 100% after the booster. To characterize the nature of the T cell responses induced by ChAdOx1-nCoV-19, PBMCs from 280 participants of phase II/III clinical trials were analyzed. Increased levels of S-specific CD4^+^ type 1 helper T cells (Th1) and CD8^+^ T cells were observed with the booster dose, but CD4^+^ Th2 cells were not detected. Both CD4^+^ Th1 and CD8^+^ responses showed a high degree of polyfunctionality in the study participants [60]. The ChAdOx1-nCoV-19 vaccine was developed by the University of Oxford and AstraZeneca and is currently in use under an emergency use authorization (EUA) in several countries.

### 2.4. ChAdOx2

ChAdOx2 is derived from ChAd68 and has E1 and E3 deletions and a modified E4 region containing E4 orf6/7 from HAd5 to increase virus yield in HEK293 cells [61]. The ChAdOx2 vector carrying four genes (AhpC, Gsd, mpa, and p12) from *Mycobacterium avium* subspecies *paratuberculosis* was found to be well-tolerated, safe, and caused a modest T cell response up to a dose of 5 × 10^10^ VP in a phase I trial [62]. Additionally, a single dose of the ChAdOx2 rabies vaccine, ChAdOx2-Rab-G, containing rabies glycoprotein (G), mounted a virus-neutralizing antibody response in mice [63].

### 2.5. Simian Ad36

The simian Ad36 has been utilized to design a vaccine for SARS-CoV-2. The ChAd-SARS-CoV-2-S bearing the SARS-CoV-2 S gene was evaluated in murine and nonhuman primate models [64,65]. Single immunization via the intranasal (i.n.) route was superior to a double dose by the i.m. route in conferring sterilizing immunity and inducing both neutralizing and mucosal IgA antibodies and a robust CMI with a high percentage of IFN-γ and granzyme-secreting T cells. In addition, immunized mice were protected from the wild type and D614G variant SARS-CoV-2 challenges [64]. Consistent with the murine model, i.n. immunization with the same vector provided protection against SARS-CoV2-challenge in rhesus macaques and limited or prevented upper and lower respiratory tract infections. The vaccine candidate was able to induce antigen-specific IgG, IgA, and neutralizing antibodies as well as antigen-specific T cells [65].

### 2.6. ChAd155

The ChAd155-based respiratory syncytial virus (RSV) vaccine was used in a phase I clinical trial in healthy adults. The replication-defective ChAd155 expressing RSV fusion (F), nucleoprotein (N), and matrix 2-1 (M2-1) proteins was well tolerated and safe at the highest dose of 5 × 10^10^ VP. It mounted a robust humoral immune response (including neutralizing antibodies) as well as IFN-γ-secreting T cells [66]. This vaccine will be assessed in phase II trials for pediatric use in RSV-seropositive and -seronegative children [67]. The immunogenicity of a ChAd155 expressing rabies G was assessed in murine and nonhuman primate models. It showed balanced humoral and CMI responses conferring protection against rabies virus challenge in mice [68].

### 2.7. PanAd3

PanAd3 was isolated from a bonobo primate. In a phase I study performed on 42 healthy adults aging from 18–50, 5 × 10^10^ VP of PanAd3 expressing three RSV antigens was given, followed by a heterologous booster of 1 × 10^7^ VP MVA expressing the same antigens using different combinations and routes including the i.m. and i.n. routes [69]. Both vectors were safe and well tolerated. There were elevated levels of antigen-specific IgG and IgA antibody-secreting cells in the i.m. prime-boost group and after i.m. boost in the i.n. primed group. Those groups showed rising RSV neutralizing antibody titer and antigen-specific IFN-γ-secreting CD8^+^ and CD4^+^ T cells. The same study was carried out on older adults aging from 60–75 and yielded similar results to the study carried out on younger adults [70].

### 2.8. ChAd7

ChAd7 has a low level of cross-reactivity with other HAds [71]. Its seroprevalence in the U.S. and China was found to be 11.8 and 8%, respectively [72,73,74]. ChAd7-based vectored vaccine candidates have been developed for many diseases, including influenza, human immunodeficiency virus (HIV), EBOV, SARS-CoV, RSV, and Zika virus [74,75,76,77,78]. In a murine model, ChAd7 carrying the M/E glycoprotein gene of Zika virus provided sterilizing immunity and eradicated viremia in mice challenged with a lethal dose. In addition, rapid cross-neutralizing antibody response and T cell response were elicited that persisted over 3 months post-immunization [74].

## 3. Bovine Ad Vectors

Bovine Ads (BAds) are primarily involved in asymptomatic to mild respiratory or gastrointestinal infections in cattle. Other clinical signs that have been associated with BAds include conjunctivitis, polyarthritis, diarrhea, and weak calf syndrome [79]. Currently, ten types of BAds are classified into two subgroups under two genera, the first subgroup includes types of BAd1, 2, 3, 9, and 10, which belong to the *Mastadenovirus* genus, and the second subgroup includes types of BAd4, 5, 6, 7, and 8 which belong to the *Atadenovirus* genus [80]. BAd3 is the most studied of the BAd types.

For foreign gene insertion, E1, E3, and E4 sites have been used. E1 and some of the E4 gene products are essential for virus replication [81]. Initially, the BAd3 vector with an E3 deletion replaced with a reporter gene was developed [81]. Subsequently, E3-deleted BAd3 vectors were used for expressing glycoprotein D of bovine herpesvirus, E2 of bovine viral diarrhea virus, and hemagglutinin of bovine coronavirus [82,83,84,85]. Cotton rats were shown to be an excellent small animal model for BAd3 and its vectored vaccines [86]. Although BAd3 vector vaccines yielded promising results in animal models [87,88], they showed low efficacy in field trials in calves due to preexisting antibodies. A BAd3 vector expressing gD of bovine herpesvirus and gG of the bovine respiratory syncytial virus was successfully generated, demonstrating that multiple antigens can be expressed in this system [89].

BAd3 internalization is independent of the HAd5 receptors, CAR, and αvβ3 or αvβ5 integrin [90] but it utilizes α(2,3)-linked as well as α(2,6)-linked sialic acids as major receptors for internalization [91]. Preexisting HAd-neutralizing antibodies in humans do not cross-neutralize BAd3 [92]. HAd-specific CMI response does not cross-react with BAd3 [93]. Unlike HAd5, BAd3 is a potent inducer of TLR4 and TLR7 [94]. There is an absence of Kupffer cell depletion with BAd3 in mice [94]; however, the Kupffer cell depletion with HAd5 is the main reason for a faster vector depletion from the host. An i.v. injection with a BAd3 vector efficiently transduces the heart, kidney, lung, liver, and spleen, and the vector persists for a longer duration compared to a HAd5 vector, especially in the heart, kidney, and lung in a mouse model [95]. Sequential administration of HAd5 and BAd3 vectors overcomes vector immunity in an immunocompetent mouse model of breast cancer [92]. The persistence of the BAd3 genome in human and nonhuman cell lines is similar to HAd5 vectors [96]. Even exceptionally high levels of HAd vector immunity do not impact the induction of humoral or CMI responses against hemagglutinin (HA) of an H5N1 influenza virus expressed in a BAd vector [97]. Therefore, the BAd3 vaccine platform offers an attractive alternative to HAd vectors for effectively immunizing individuals with high levels of preexisting HAd immunity with safety aspects similar to HAd5 vectors.

Immunogenicity and efficacy of protection conferred by a BAd3 vector-based H5N1 influenza vaccine were significantly better than those of the HAd5 vector-based H5N1 vaccine [88]. This level of antigen-specific enhancement of humoral and CMI responses resulting in complete protection at a 30-fold lower dose [88] strongly signifies the usefulness of the BAd vector platform for developing an effective influenza pandemic vaccine. Recently, a BAd-based tuberculosis vaccine expressing the mycobacterial Ag85B-p25 epitope and the autophagy-inducing peptide C5 (AIP-C5) of *Mycobacterium tuberculosis* (Mtb) CFP10 protein was evaluated [98]. This vector can better process the antigen in APCs through autophagy and presentation of the Ag85B-p25 epitope to CD4^+^ T cells. Mice immunized i.n. showed significant protection following aerosolized challenge with Mtb, either alone or as a booster after the bacille Calmette–Guerin (BCG) vaccine. In addition, there was considerable expansion of CD4 and CD8 central memory (T_CM_), effector (T_EM_), and resident memory (T_RM_) T cells in the lungs of immunized mice.

## 4. Canine Ad Vectors

Canine Ad (CAd) vectors based on both type 1 (CAd1) and type 2 (CAd2) were developed, but the latter is being utilized for vaccine development [99]. The E1-deleted CAd2 can only be propagated in canine kidney cells expressing CAd1 E1 gene products and has an insertion capacity of up to ~4kb of the transgene cassette [100]. An E3-deleted replication-competent CAd2 carrying rabies virus G glycoprotein, CAdV-2-RVG, has been evaluated for immunogenicity and protection efficacy in mice, dogs, cats, pigs, and sheep [101,102,103,104,105]. Immunized animals were protected following a rabies virus challenge. Both replication-defective (Cav-G R^0^) and replication-competent vectors (Cav-G R^+^) carrying the rabies virus G glycoprotein gene were utilized to immunize sheep in a single dose regimen either via the i.m. or subcutaneous (s.c.) route [103]. Both vectors elicited neutralizing antibodies by either route of administration, but Cav-G R^+^ showed improved stimulation of antigen-specific IFN-γ-producing CD4^+^ and CD8^+^ T cells.

A CAd2 vector-based vaccine, Cav-P1/3C R°, expressing the foot-and-mouth disease virus (FMDV) strain O/FRA/1/2001 structural P1 precursor protein along with the nonstructural 3C protein was evaluated in guinea pigs for immunogenicity and protective efficacy [106]. The vaccine mounted a robust humoral response in immunized animals and provided heterologous protection against generalized FMD following FMDV strain O1 Manisa/Turkey/1969 challenge. In addition, a replication-competent CAd2 vector bearing the rabbit hemorrhagic disease virus (RHDV) vp60 gene, rCAV2-VP60, was used to immunize 4-month-old Japanese big-ear white rabbits by the i.m. route with a dose of 10^7^ tissue culture infective dose 50 (TCID_50_) twice at 2 weeks apart [107]. Following the booster vaccination, a robust humoral response conferred protection against challenge with RHDV-HYD isolate. Moreover, a replication-competent CAd2 vector, CAV-2-ROP18, carrying the ROP18 gene of *Toxoplasma gondii* resulted in a balanced immune response in mice characterized by mixed IgG1/IgG2a antibodies against ROP18, INF-γ-producing T cells that displayed a Th1 biased helper T cell profile [108]. In addition, immunized mice were partially protected (40% survival) against a lethal challenge with the *T. gondii* RH strain (genotype II). Collectively, these results demonstrate the potential of CAd vectors in developing vaccines against infectious diseases of both humans and animals.

## 5. Porcine Ad Vectors

Five types of porcine Ad (PAd), PAd1–5, have been isolated from pigs with asymptomatic to mild intestinal or respiratory infections [80]. However, the genomic structure of PAd3 is similar to HAd5 but did not show cross-neutralization with HAd-specific neutralizing antibodies [94]. Additionally, the PAd3 vector can transduce the human, bovine, murine, and porcine cells in culture; thus, it offers a promising alternative or supplement to HAd vectors [109].

The E1, E2, E3, and E4 transcription regions can be used for developing PAd3 vectors for gene delivery [110,111,112]; however, the E3 region is widely used. PAd3 was first used to deliver the gp55 of the classical swine fever virus (CSFV) gene without any deletion in the PAd3 genome [113]. Outbred pigs were inoculated s.c. with a single dose of the recombinant PAd3 vaccine containing the gp55 gene, resulting in a robust immune response comprising a high level of gp55-specific antibodies and complete protection from a lethal challenge with CSFV. A DNA vaccine containing the same gp55 protein from CSFV was administered to 6-week-old weaned pigs and then boosted with recombinant PAd3 vaccine in a prime-boost strategy, resulting in high antibody levels conferring 100% protection after the challenge with CFSV [113]. Later, another vaccine was developed by inserting the gD gene of pseudorabies virus in the E3 region of PAd3 to vaccinate pigs [114]. In another study, an orally administrated PAd5 vector-based vaccine expressing S glycoprotein of swine transmissible gastroenteritis virus in the E3 region was developed [115]. A surveillance study showed that 90% of pigs had preexisting antibodies against PAd3 [116]. Interestingly, preexisting vector immunity did not adversely affect the efficacy of the PAd3-based CSFV vaccine [117,118], suggesting that PAd3 vectors can be utilized as a potential gene delivery system for its natural host. However, considering obvious safety issues, replication-defective PAd3 vectors will be better for species other than swine [119].

The E1 region of PAd3 is similar to HAd5 [118]; therefore, E1-complementing cell lines were developed [118,120] with diverse tropism and future use for generating novel targeted vectors. A PAd3-based influenza vaccine (PAd3-HA) expressing HA of an H5N1 influenza virus was evaluated in mice [121]. Immune responses and protection efficacy were evaluated following immunization with different doses of PAd3-HA or HAd5-HA. The PAd3-HA vaccine group displayed a superior survival rate with a lower virus load compared to the HAd5-HA group. The long-term evaluation demonstrated that PAd3-HA conferred better protection than HAd5-HA at 12 months post-vaccination. It is important to note that PAd3 showed no significant neutralization by human serum samples [109,121]. Overall, it seems that the PAd-based vector system is a viable and desirable supplement to the HAd-based delivery vehicles.

## 6. Ovine Ad Vectors

Seven distinct types of Ad are isolated from sheep, referred to as ovine Ad types 1–7 (OAd1-7), and six of them are found in the genus *Mastadenovirus* (OAd1-6), while OAd7 belongs to the genus *Atadenovirus* [122]. OAd7 harbors distinct biological and structural features that differentiate it from the *Mastadenoviruses*, as it carries two structural genes and many nonstructural genes that are unique to *Atadenovirus*. OAd7 also uses a receptor different from CAR and lacks the integrin-binding domain on its penton base protein [123,124,125]. Moreover, OAd7 is attenuated for human cells and can carry up to a 6.3 kbp transgene cassette. There are three nonessential sites in the OAd7 genome referred to as sites I, II, and III for foreign gene insertion. Site I is located between the viral fiber gene and protein VIII gene, while site II is situated towards the viral genome’s right end. Site III is situated between E4 and the right end transcription unit and is by far the most preferred site for insertion, as inserts at this site are stable for 10–15 passages [123]. Several OAd7 vectors expressing immunogenic transgenes have been generated [126,127,128,129].

The OAd7 vector expressing HCV NS3 protein was evaluated in mice via the i.m. route of immunization, inducing robust and long-lasting antigen-specific T cell response [128]. An OAd7-based HIV-1 vaccine candidate expressing an HIV-1 clade A consensus gag-derived protein coupled to a T cell polyepitope was evaluated in mice alone or a heterologous prime-boost regimen with HAd5 or MVA expressing the same antigen [126]. The OAd7-based vaccine alone or in combination was highly immunogenic, and induced polyfunctional HIV-1-specific T cell responses, and reduced viral replication by 3–4-fold. Notably, the effect was not impacted by preexisting anti-HAd5 immunity.

## 7. Avian Ad Vectors

Ads have been known to infect several birds, including fowl, geese, ducks, falcons, and turkeys. Most of the viruses isolated from birds fall under the genus *Aviadenovirus* and are further classified into the following types: fowl (FAd A–E), goose (GoAd A), duck (DAd A), and turkey (TAd B). These avian Ads (AAds) are considered serologically distinct from other members of Ad genera [130,131]. The genomes of AAds are significantly larger, have higher GC contents, and lack the genes that encode E1, E3, V, and IX compared to other Ads [130,132].

FAd vectors are the most commonly used among AAds. FAdV-based vaccines have been developed using homologous recombination techniques and evaluated for their immunogenicity and protection efficacy. A FAd type 10 (FAd10) vector expressing the infectious bursal disease virus (IBDV) VP2 gene under the control of the FAd10 major late promoter/leader sequence (MLP/LS) was grown in kidney cells derived from 3-week-old specific pathogen-free chickens (SPFC) [133]. Chickens vaccinated i.v., intraperitoneally (i.p.), or s.c. with FAd10/VP2 showed VP2-specific antibodies that provided protection against challenge with a virulent IBDV V877 strain [133]. The S1 subunit of the spike protein of avian infectious bronchitis virus (IBV) was used to develop a FAd8-based vaccine [134]. Immunized chickens showed S1-specific antibody and CMI responses and were protected from challenge with either Vic S or N1/62 strains of IBV [134].

## 8. Conclusions and Future Directions

Nonhuman Ad vectors were developed to overcome preexisting HAd immunity and provide versatility in receptor usage, cell tropism, and induction of antigen-specific immune responses. It has now become apparent that HAd-neutralizing antibodies do not significantly cross-neutralize nonhuman Ads. Still, they share many features of HAds in cell transduction and the development of balanced humoral and CMI responses.

The nonhuman Ad vector platform primarily based on ChAd has attracted substantial interest in recent years, exemplified by a growing number of preclinical and clinical studies for evaluating effective vaccines for several infectious diseases. The development and use of the Oxford/AstraZeneca ChAd-based SARS-CoV-2 vaccine in millions of people in several countries have been a landmark elucidating the safety and potential concerns of nonhuman Ad-based vaccines for human and veterinary applications. It has been reported that the Oxford/AstraZeneca ChAdOx1 nCoV-19 vaccine was associated with an increased incidence of immune thrombocytopenic purpura (ITP), leading to the formation of blood clots in a few persons with a history of severe allergic reactions. In a recent study, five cases of ITP were reported in more than 130,000 ChAdOx1 nCoV-19-vaccinated individuals [135]. Now, ITP cases can easily be handled using routine treatment options. Individuals with a history of severe allergic reactions should not receive the Oxford/AstraZeneca COVID-19 vaccine. It is unclear whether ITP is induced by the ChAd vector or S protein since ITP is also observed in people with COVID-19 infections or following immunization with an mRNA vaccine [136]. Overall, the immunization of millions of people with the ChAdOx1 nCoV-19 vaccine has demonstrated the acceptability of nonhuman Ad vectored vaccines for humans. The longevity of vector-specific neutralizing antibodies in people vaccinated with an Ad vector-based COVID-19 vaccine will determine whether annual immunization with the same Ad vector will be feasible without impacting the quality of protective immune responses. Our study to determine the longevity of vector immunity and its impact on the resultant antigen-specific immunity in mice implies that yearly vaccination with the same vector may be possible [137]. If an acceptable decline in vector-specific neutralizing antibodies within a year is not achieved, we will be better off using a different Ad vector-based vaccine for effective protection. Currently, we have a range of nonhuman and rare human Ad vectors to choose from for subsequent immunization needs.

Despite the current progress, further research is needed to ascertain the safety concerning the interaction with blood clotting mechanism/s and examine the persistence of a nonhuman Ad genome in the host. The durability of protective immunity generated following the first use of a nonhuman Ad vectored vaccine will be vital for deciding the time frame for successful booster immunization. The use of nonhuman and human Ad vectors or two nonhuman Ad vectors for the prime-boost approach will enhance the level and longevity of antigen-specific protective immune responses.

Increased understanding of the biology of nonhuman Ads and mechanism/s of antigen processing in generating a durable protective immune response will undoubtedly help in designing the next generation of nonhuman Ad-based vaccines. The certification of cell lines for cGMP production of other nonhuman Ad vector-based vaccines will be necessary for evaluating their efficacy in human trials. In addition, exploring cellular tropism and receptor utilization of several other nonhuman Ad vectors is still needed to exploit their full potential.

## Figures and Tables

**Table 1:** Key properties of various nonhuman adenoviral vectors for vaccine delivery.

Prototype Strain of Non-Human Adenovirus	Max Transgene Insertion Capacity	Deletion/Insertion Sites	Primary Receptor Utilized	Cell Line Used for Propagation
Simian adenovirus type 25	~8 kb	E1 and E3 regions	CAR	HEK293 cells or any HAd5 E1 transformed cell lines
Bovine adenovirus type 3	~5.5 kb	E1 and E3 regions	*α*(2,3)-linked and *α*(2,6)-linked SA	Bovine-human hybrid cells (BHH3 and BHH8); BAd3 E1 complementing cell lines; or HAd5 E1 transformed fetal bovine retinal cells (VIDO R2 and FBRT HE1)
Canine adenovirus type 2	~4.0 kb	E1 and E3 regions	CAR	CAd2 E1 transformed DK cells
Ovine adenovirus type 7	~6.3 kb	Site I: pVIII and fiber intergenic region; Site II: unique *SalI* site within ORF RH2; & Site III: region between the putative E4 transcription units and the right end	INT	Ovine Fetal skin fibroblast producer cell line (HVO156) or Sheep fetal lung cells (CSL503)
Porcine adenovirus type 3	~4.7 kb	E1 and E3 regions		HAd5 E1 transformed porcine fetal retinal cell lines (VIDO R1 and FPRT HE1-5)
Avian adenovirus (Fowl adenovirus type 1)	~4.0 kb	Region between E4 promotor and right ITR; Region between 938 and 2900 (requires trans-complementation); and Three ORFs adjacent to right end of the genome	CAR	Leghorn male hepatoma (LMH) cell line

CAR, Coxsackievirus and adenovirus receptor; SA, sialic acid; INT, integrins; ORF, open reading frame.

## Data Availability

Not applicable.
